# The evolution of prey-attraction strategies in spiders: the interplay between foraging and predator avoidance

**DOI:** 10.1007/s00442-023-05427-5

**Published:** 2023-08-04

**Authors:** Tom Ratz, Julien Bourdiol, Stéphanie Moreau, Catherine Vadnais, Pierre-Olivier Montiglio

**Affiliations:** 1 Behavioural Ecology, Department of Biology, Ludwig-Maximilians-Universität in Munich, 82152 Planegg-Martinsried, Germany; 2 Département des Sciences Biologiques, Université du Québec à Montréal, CP-8888 Succursale Centre-ville, Montréal, QC H3C 3P Canada

**Keywords:** Araneae, Lure, Phenotypic variation, Sensory ecology, Trade-off

## Abstract

Lures and other adaptations for prey attraction are particularly interesting from an evolutionary viewpoint because they are characterized by correlational selection, involve multicomponent signals, and likely reflect a compromise between maximizing conspicuousness to prey while avoiding drawing attention of enemies and predators. Therefore, investigating the evolution of lure and prey-attraction adaptations can help us understand a larger set of traits governing interactions among organisms. We review the literature focusing on spiders (*Araneae*), which is the most diverse animal group using prey attraction and show that the evolution of prey-attraction strategies must be driven by a trade-off between foraging and predator avoidance. This is because increasing detectability by potential prey often also results in increased detectability by predators higher in the food chain. Thus increasing prey attraction must come at a cost of increased risk of predation. Given this trade-off, we should expect lures and other prey-attraction traits to remain suboptimal despite a potential to reach an optimal level of attractiveness. We argue that the presence of this trade-off and the multivariate nature of prey-attraction traits are two important mechanisms that might maintain the diversity of prey-attraction strategies within and between species. Overall, we aim to stimulate research on this topic and progress in our general understanding of the diversity of predator and prey interactions.

## Introduction

Traits mediating interactions among species are central in explaining the structure of ecological communities (Wootton 1994; Berlow 1999; Werner and Peacor 2003) and variation in major life-history events (e.g., growth, fecundity, and mortality) within and between species (Thompson 1999). This is particularly the case between predators and prey, because the traits mediating predator–prey interactions such as attack rate and predator avoidance often are key mechanisms structuring ecological communities (Werner and Peacor 2003; Schmitz et al. 2004). Theory predicts that natural selection should act towards stabilizing such dynamics and eventually settling predator–prey interactions to stable predator and prey phenotypes (e.g., Hochberg and Holt 1995, Loeuille 2010, but see Matsuda and Abrams 1994, Abrams 2000). There is, however, ample empirical evidence and theoretical literature showing that phenotypes with an important role in predator–prey interactions often show higher variation both between and within individuals of the same species (Bolnick et al. 2003, 2011; Saloniemi 1993; Doebeli and Koella 1994; Okuyama 2008). This presents an ecological puzzle. How can we explain that variation in traits mediating predator–prey interactions is maintained over the course of evolution? And, in turn, what prevents predator–prey dynamics from reaching a stable equilibrium (Hendry 2016, Chap. 8)?

One possible explanation for the maintenance of such variation is the existence of evolutionary trade-offs involving multiple traits (DeWitt and Langerhans 2003; Langerhans 2007; Peiman and Robinson 2017). For instance, there is a trade-off between foraging ability and reproductive success if predators have limited energy and time to allocate between these two functions (e.g., Yeh et al. 2015; Fan et al. 2009; Lima 1998a, b). The trade-off between foraging effort and predator avoidance should be particularly important in species that attract their prey using lures and are relatively low in the food chain, such as mesopredators (hereafter non-apex predators; Yeh et al. 2015). This is because non-apex predators that attract prey both face greater risk of predation from species higher up in the food chain (that are not the target of lures) by drawing their attention. Although an extensive number of empirical studies have documented prey-attraction strategies in non-apex predators (Box 1), the evolution of lures has received little comprehensive treatment. Attracting prey should come at a cost of increased vulnerability to predators that occupy the same or a higher rank in the food chain (see Magnhagen 1991 and Zuk and Kolluru 1998 for a similar concept in a mating context). Thus, animals using lures might need to balance foraging success with predation risk, both of which increase with the use of more conspicuous lures. Predators using lures might produce conspicuous signals to attract their prey that are, in turn, exploited by their own predators to locate them. This, however, may not always be the case as research on toxicity signals in prey (i.e., aposematism) has claimed that the emergence of conspicuous signals may paradoxically be possible as a result of relaxed predation in early evolutionary phases (Marples et al. 2005; Mappes et al. 2005). The existence of a trade-off between prey attraction and predator avoidance is, thus, debatable.

In this synthesis, we review the literature on prey-attraction adaptions in spiders (Araneae). This group contains arguably the largest number of species using prey attraction as their main foraging mode. Spiders represent an ideal study group to explore the trade-off between prey attraction and predator avoidance for three main reasons. First, spiders are predators that are low in the food chain and hence have many potential predators themselves (e.g., vertebrates predators, parasitoid wasps, other spiders; Wise 1993). Second, spiders are a species-rich group with over 51,000 species (World Spider Catalog 2023), exhibiting diverse life-histories and foraging strategies with numerous taxa using prey-attraction strategies. Third, silk usage and web production allow a diversity of structure and extends the foraging potential of this group: spider silk and webs can be used in addition to, or in place of, the spider's own body to attract prey. Using lures made of silk should also be important in reducing mortality due to predation: instead of targeting directly the spider, predators might instead target silk structures, that would, thus, serve as “decoys” and contribute to reduce the spider’s risk of death.

We first provide an overview of prey-attraction strategies in animals, with a particular focus on spiders. We next review the evidence for a trade-off between prey attraction and predator avoidance in spiders and emphasize the importance of sensory mechanisms that underlie the trade-off. We discuss the potential scenarios governing the evolution of lures and stress the importance of intraspecific variation and phenotypic plasticity in prey-attraction strategies in shaping predator–prey coevolution. Our objective is to provide a first account of the topic, highlight general patterns, and provide directions for future empirical work.

## Adaptations to attract prey in spiders

A diversity of spider taxa have developed highly visible phenotypes for attracting prey (Blackledge et al 2011; Walter and Elgar 2012; Chap. 8 in Stevens 2013) through a) the addition of a visual signal to their web, in the form of silk threads or other material (hereafter “web decorations”), b) conspicuous body coloration, or c) attractive volatile chemical compounds and vibratory signals. In this section, we review each of these types of traits.

### Web decorations

Many species of spiders, particularly species belonging to the genus *Argiope* (Herberstein et al. 2000; Cheng et al. 2010; Walter and Elgar 2012), decorate their web with additional threads of silk. These web decorations (also known as stabilimenta) are made of tightly woven silk, shaped into different patterns near the center of the web and the spider (Bruce et al. 2001). Such web decorations are highly visible and do not seem to reinforce the structure of the web (Simon 1864; Eberhard 1990; Herberstein 2000; Starks 2002; Bruce 2006; Théry and Casas 2009; Blackledge et al. 2011). Studies conducted under both natural and laboratory conditions show that web decoration often increase the number of prey drawn in and caught in the web by making the web more attractive (Craig and Bernard 1990; Craig 1991; Craig and Ebert 1994; Tso 1996; Tso 1998a,b; Watanabe 1999; Herberstein 2000; Bruce et al. 2001; Bruce et al. 2004; Cheng and Tso 2007; but see Hauber 1998). Web decorations presumably attract insect prey to the spider web either because they resemble light gaps in the vegetation (Elgar et al. 1996; Ewer 1972) or because their pattern of UV light reflectance mimics that of flowers (Craig and Bernard 1990; Craig 1995; Kiltie 1996). This white structure could in fact reflect many wavelengths and might, thus, be capable of reaching longer distances relative to monochromatic structures: as distance increases, the number of wavelengths that are scattered or absorbed increases and, thus, a more diverse spectrum (i.e., white light) is more likely to be visible from further away (Endler 1992; Manning and Dawkins 1998).

Alternatively, web decorations could help to protect the spider against predators and parasitoids (Marples et al. 2005; Horton 1981; Eisner and Nowicki 1983; Schoener and Spiller 1992; Kerr 1993; Cloudsley-Thompson 1995; Blackledge 1998b; Blackledge and Wenzel 1999; Nakata 2009). Web decorations may conceal the spider (Hingston 1927; Bristowe 1941; Ewer 1972; Eberhard 1973, 1990; Tolbert 1975; Edmunds and Edmunds 1986; Schoener and Spiller 1992; Tan and Li 2009; Wang et al. 2021), make it appear bigger (Hingston 1927; Ewer 1972; Eberhard 1973; Tolbert 1975; Schoener and Spiller 1992; Li and Lee 2004; Uhl 2008), act as a retreat (Blackledge and Wenzel 2001; Walter 2018), or physically shield the spider from attacks (Tolbert 1975; Schoener and Spiller 1992; Blackledge and Wenzel 2001). Web decorations can also prevent parasitoid wasps from identifying and accessing the spider (Blackledge and Wenzel 2001). More generally, web decorations can reduce the risk of damage to the web caused by flying birds by signaling the presence of the web and preventing accidental bird fly-through (Horton, 1980; Eisner and Nowicki 1983; Blackledge and Wenzel 1999). For example, Blackledge and Wenzel (1999) found that webs without decorations were more often damaged by birds.

Nevertheless, it is clear that web decorations often attract prey and increase foraging success (Table 1) and several studies suggest that this might come with an increase in predation risk (Bruce et al. 2001, 2005; Seah and Li 2001; Li and Lee 2004). For example, Li and Lee (2004) found that *Argiope* spiders were less likely to build and, when they did, built smaller web decorations in response to the presence of olfactory cues from a predator. There is also evidence that predators are able to detect web decorations and memorize their form (Seah and Li 2001). This could ultimately lead to an increase in the risk of predation associated with the use of web decorations.

**Table 1:** Empirical studies testing for a trade-off between prey attraction and predator avoidance in spiders

References	Species	Lure	Type of experiment	Trade-off	Function
Blakcledge and Wenzel (1999)	*Argiope aurantia*	Web decoration	Field	Yes	Decreased prey capture and damage from birds
Bruce et al. (2001)	*Argiope keyserlingi*	Web decoration	Field and laboratory	Yes	Increased prey capture and predation rate
Cheng and Tso (2007)	*Argiope aemula*	Web decoration	Field	Yes	Increased prey capture and predation rate
Craig et al. (2001)	*Argiope argentata*	Web decoration	Field and laboratory	Likely	May increase prey capture and predation rate
Fan et al. (2009)	*Nephila pilipes*	Body coloration	Field	Yes	Increased prey and predator attraction
Heiling et al. (2005a)	*Thomisus spectabilis*	Body reflection	laboratory	Likely	May increase prey capture and predation rate
Nakata (2009)	*Cyclosa argenteoalba*	Web decoration	laboratory	Unlikely	May reduce predation rate but not prey capture
Tan and Li (2009)	*Cyclosa mulmeinensis*	Detritus decoration	Field	Likely	Reduced visibility from both prey and predators
Yeh et al. (2015)	*Argiope aemula*	Web decoration	Field	Yes	Increased prey and predator attraction

### Body coloration

Some spiders also exhibit conspicuous body coloration or markings that attract prey (e.g., White 2017; Hauber 2002; Chuang et al. 2007; Tso et al. 2007). For example, body coloration increases foraging success in the golden orb-weaver spider *Nephila pilipes* and the spotted orb-web spider *Neoscona punctigera* (Chuang et al. 2007; Chiao et al. 2009; Blamires et al. 2012), the araneomorph spider *Psechrus clavis* (Lai et al. 2017), and the northern jeweled spider *Gasteracantha fornicata* (Muma 1971; Hauber 2002; White and Kemp 2016; White 2017). In the genus *Gasteracantha*, females exhibit bands of bright color, generally white or yellow, that contrast against a black outline and lure prey to the web (Hauber 2002; Rao et al. 2015; White and Kemp 2016; Messas et al. 2021). Likewise, the crab spider *Epicadus heterogaster* uses its abdomen, which reflects UVs, to attract prey (Vieira et al 2017). The brown huntsman spider *Heteropoda venatoria* bears a white stripe on its forehead that attracts flying prey at night, such as moths (Zhang et al 2015).

### Non-visual lures

Spiders also attract prey using non-visual lures, such as chemical and vibratory signals. For example, Bolas spiders of the genus *Mastophora* attract male moths by producing volatiles that mimic female moth pheromones (Eberhard 1977; Stowe et al. 1987; Haynes et al. 2002; chap. 11 in Nentwig 2013; chap. 8 in Stevens 2013). The St Andrew's cross spider *Argiope keyserlingi* mists its webs with the amide putrescine, which serves as an allomone that increases the rate of prey capture (Henneken et al. 2017a). The social spider *Mallos gregalis* attracts flies using the odor produced by yeasts that grow on the carcasses of flies which the spider aggregates in the web (Tietjen et al. 1987). The jumping spiders of the genera *Brettus*, *Cyrba*, *Gelotia*, and *Portia* prey on other spiders by attacking them on their webs using vibrations that mimic a prey caught in the web (Jackson and Blest 1982; Jackson and Hallas 1986; Jackson 1990a, b, 1992). Outside these examples, non-visual lures such as volatile compounds and vibratory signals are extremely understudied compared to visual ones (i.e., web decorations and conspicuous body coloration). In spiders, only a handful of studies have studied olfactory and tactile lures, with most studies focusing on chemical lures of bolas spiders (Zhu and Haines, 2004; Vereecken and McNeil 2010). Although non-visual lures might be widespread among spider taxa (Uetz and Roberts 2002; Hill and Wessel 2016; Virant-Doberlet et al. 2019), we currently have limited understanding of whether they are common hunting tactics in spiders or if they involve more complex strategies using multimodal mechanisms in combination with chemical and/or vibratory signals.

## The trade-off: catching prey versus avoiding predators

There is good evidence that predator foraging behavior and prey anti-predator traits can coevolve (Dawkins and Krebs 1979; Abrams 2000), and that there is often a trade-off between maximizing foraging success and avoiding predation (e.g., Lima 1998a, b; Houston et al. 1993). For example, foraging efficiency decreases under greater risk of predation in back-swimmers (Sih 1980), marmots (Holmes 1984), and chickadees (Lima 1985). Likewise, lures are most likely shaped by both top-down and bottom-up selective pressures, and we should expect to see evidence of a trade-off maximizing prey capture while limiting predation risk (Blackledge 1998a; Yeh et al. 2015; Fan et al. 2009). Past studies in arachnids have investigated this potential trade-off mainly in visual signals, with web decorations being the most documented cases. Interestingly, web decorations can also increase the risk of predation by attracting more insect predators, such as mantids (Bruce et al. 2001) and wasps (Cheng and Tso 2007). This suggests that spiders using web decorations might face a trade-off between prey attraction and predator avoidance.

There is also some evidence for a trade-off between prey attraction and predator avoidance in other species using visual signals to attract their prey. For example, Fan et al. (2009) tested the attracting properties of the black and yellow pattern of the orb-web spider *Nephila pilipes* and showed that yellow coloration attracts both predators and prey. This suggests that the common bright-and-dark coloration could be an optimal phenotype negotiating the trade-off between prey and predator attraction. In contrast to visual lures, we are not aware of any studies reporting similar effects of chemical or vibratory lures attracting predators. It is, therefore, unclear whether non-visual lures could also be constrained by a trade-off between prey attraction and predator avoidance.

Given that variation in the abundance of resources and predation risk are major evolutionary drivers shaping natural populations (Sih et al. 1985; Lima 1998b; Langerhans 2007), the trade-off between foraging success and predation avoidance might play an important role in maintaining variation in the extent to which spiders build web decorations and express body coloration (Cheng and Tso 2007; Fan et al. 2009). The members of the *Argiope* genus, for example, sometimes reduce or even stop decorating their web when facing a greater predation risk. In some cases, these spiders rely solely on their body coloration as lure (Cheng and Tso 2007). Part of this variation over time might come from reversible phenotypic plasticity. The expression of lures could be condition dependent: the investment made by individuals into their lures varies with diet or past foraging success (Heberstein et al. 2000). Indeed, Gawryszewski et al. (2012) found that past foraging success was related to color contrast between spiders and their background in the crab spider *Thomisus spectabilis*.

Although the existence of a trade-off between prey attraction and predator avoidance is expected and supported by some empirical work, there currently is no clear consensus as to whether lures really are associated with increased costs of predation. Research on sexual selection and aposematism (i.e., signaling mating benefits or toxicity) have debated similar issues and provide useful explanations on how conspicuous signals can evolve and become fixed in populations. In the context of signaling among sexual partners, deploying conspicuous signals should increase detectability by predators (reviewed in Burk 1982; Magnhagen 1991; Zuk and Kolluru 1998; Haynes and Yeargan 1999; Kotiaho 2001) and there should, therefore, be a trade-off between signaling and predator avoidance. Yet there is not always an obvious predation cost associated with sexual signals (White et al. 2022), potentially because more conspicuous individuals tend to also express stronger anti-predator behavior to compensate for their increased visibility to predators (Bernal and Page 2023). This would be the case, for instance, if individuals in better condition or that have access to more resources can invest more in both sexual signals and anti-predator behavior, whereby masking the potential trade-off between signaling and predator avoidance (Van Noordwijk and De Jong 1986).

The presence of a trade-off is even more controversial in the context of aposematism, where toxic prey develop conspicuous visual signal to warn predators of their toxicity. To associate an aposematic prey signal with toxicity, predators have to initially learn by consuming aposematic prey (Guilford 1988). Hence, any new prey variant displaying a conspicuous signal would likely be rapidly purged out of the population before being established (Guilford 1990). This apparent paradox is resolved by empirical studies showing that predators often avoid new prey items as a result of neophobia or dietary conservatism (Mappes et al. 2005; Marples et al. 2005; Crane and Ferrari 2017; White and Umbers 2021). Theoretical work has also suggested that predators might simply avoid conspicuous prey if it means that such prey are more likely to have been encountered by other predators and survived and are, thus, more likely to be toxic (Sherratt 2002). Regardless of the specific explanation, it is clear that in the case of aposematism increased detectability to predators does not necessarily imply increased predation. Nevertheless, it is unlikely to be the case in situations where the prey signals are not intended for predators, but are instead produced to achieve another function such as prey attraction, and are, incidentally, exploited by eavesdropping predators. Although neophobia and dietary conservatism could enable variants expressing a new prey-attraction phenotype to survive and initially spread in a population, it seems unlikely that this mechanism would allow such variants to become established in the long term, when generations of predators and prey have been sharing the same environment. The reason is because, unlike aposematism where the signal can be reinforced when established because prey are toxic, lures and prey-attraction tactics do not represent a problem for predators that have learned to identify them.

Another reason that could explain the absence of a trade-off is if there is a convergence in the signals attracting prey and warning predators. Spiders may also have developed tactics to avoid predation. For example, signalers in the context of sexual selection often mitigate predator attraction using “private” signals detectable by conspecifics but not by predators (e.g., Endler 1992; Cummings et al. 2003), or by adjusting the timing or location of signaling (Bernal and Page 2023). For example, fireflies use flashing signals instead of constant glow to reduce the risk of predation (Lloyd 1983), while males in Blue-black grassquits tend to display their iridescent plumage in direct sunlight only to maximize conspicuousness and avoid displaying a signal continuously (Sicsú et al. 2013). In the context of prey attraction, it is, thus, possible that spiders modulate the display of lures depending on the risk of predation. Some signals could also achieve two functions: attracting prey and signaling toxicity or unpalatability to predators. This may be the case for *Gasteracantha* spiders that display a conspicuous body coloration attracting prey while at the same time bearing striking morphological defences. In this species, body coloration could serve as signal to lure prey *and* as aposematic signal to warn predators (Gawryszewski and Motta 2012; Ximenes and Gawryszewski; 2019).

Nevertheless, the existence of a trade-off between prey attraction and predator avoidance has seldom been tested and there is a need for more studies addressing this pressing issue. Such studies should aim at estimating the predation costs associated with prey attraction by, for example, experimentally manipulating the characteristics of lures (Bruce et al. 2001). This approach would be valuable to reveal the presence of a trade-off, which should become apparent if manipulations that increase prey attraction also increase the rate of predation. It would also help identify characteristics of lures that play a key role in attraction and that might be under strong selective pressure imposed by predators and prey. Moreover, there is a bias in studies towards visual lures used in prey attraction (Table 1) and more research is needed on non-visual lures in species that use chemical or vibratory lures. We suggest that the next logical step will be to improve our understanding of this trade-off by analyzing the effect of lures on predation itself and dissecting the mechanisms underlying the evolution of lures to pinpoint the exact mechanical or physiological constraints underlying the possible trade-off and determining how individual spiders negotiate it. In the next section, we present the most relevant mechanisms in our opinion.

## Sensory adaptations to multiple selective pressures

The sensory mechanisms underlying lures remain poorly understood. Lures must be under selection to respond to constraints at multiple levels, such as environmental conditions (e.g., ambient light, prey abundance, predator presence), the physical properties of lures (e.g., color brightness, reflectance), and the sensory capabilities of prey (e.g., photoreceptors and neural processing of signals; White and Kemp 2015). Studies investigating the properties of visual signals used in prey attraction (body coloration and web decorations) show that lures often rely on contrasts (from colors to shades of black and white) and light reflection (UV reflection; White and Kemp 2016; Chiao et al. 2009). Therefore, the evolution of lures involves the coevolution of multiple aspects of a trait to form a multivariate phenotype. This aspect is rarely considered by studies focusing on one or two aspects at a time. The combination of UV reflectance, chromatic and achromatic properties has been studied the most (White and Kemp 2016; Heiling and Herberstein 2004; Bruce and Heberstein 2005; Chiao et al. 2009), but these might not be the only ones.

Lures are complex signals under diverse selection pressure that might often involve a composite of multiple traits. Such multicomponent signals are characterized by the use of additional features to reinforce a main signal (Partan and Marler 2005; Higham and Hebets 2013) and have been mainly studied in spiders in the context of sexual communication (e.g., Rypstra et al. 2009). The use of lures to attract prey should also involve multicomponent signals given that prey generally use a combination of cues to identify potential food sources while avoiding predation (Llandres et al. 2011). As such, spiders that use lures combining multiple types of attractive signals, such as symmetry (White and Kemp 2020) or body position (Cheng, Heiling, and Heberstein 2006), should achieve a greater attractiveness. Other visual aspects are likely to play a role as well, such as form-related aspects, including shape, angle of vision, and size (Cheng et al. 2010). In addition to shape, brightness and geometric patterns can contribute to attract prey, such as in the northern jeweled spider *G. fornicata* (White 2017; White and Kemp 2016).

To successfully attract prey, spiders have to produce lures that are adjusted to the sensory system of their prey. Luring spiders achieve this by taking advantage of pre-existing biases in their prey’s sensory system and preference for particular stimuli (e.g., foraging preferences, White and Kemp 2020; mating behavior, De Serrano et al. 2012). For visual lures to effectively deceive prey, spiders must manipulate multiple visual aspects such as color contrasts, achromatic contrasts, and light reflection (White and Kemp 2016; Chiao et al. 2009). Spiders that mimic flowers to attract pollinators provide an enlightening demonstration of the multiple constraints that prey sensory preferences impose (White and Kemp 2016). In these spiders, the lure mimics the color patterns, shapes, contrasts, luminance, and symmetry of a flower (White and Kemp 2020, 2017; Vieira et al. 2017; Chiao et al. 2009; Cheng et al. 2010). Such an elaborate mimicry enables the crab spider *Epicadus heterogaster* to successfully attract prey by using its flower-shaped, UV-reflecting abdomen that seems equally attractive to pollinators as real flowers (Vieira et al. 2017).

In turn, the sensory system of local prey and their response to lures evolve as a result of selection exerted by spiders. For example, the Australian native bee *Austroplebia australis* avoids flowers occupied by local crab spiders, whereas the introduced bee *Apis mellifera* is unable to discriminate between safe and risky flowers (Heiling and Heberstein 2004). This is likely due to a change in the sensory perceptions and preferences of native bees that have a shared evolutionary history with local crab spiders and, as a result, have been selected to identify and avoid their lures (Heiling and Heberstein 2004). Such coevolutionary dynamics linking prey preference and spider lures have also been documented among *Argiope* spiders using web decorations, for which the shape presumably evolved from linear to cross-like to meet the symmetry preferences of potential insect prey (Cheng et al. 2010).

The trade-off between prey attraction and predator avoidance should also be important in shaping the evolution of sensory adaptations. Indirect empirical evidence suggests that conspicuous signals are vulnerable to eavesdropping from predators because sensory capabilities and preferences of prey and predators often overlaps. For example, in many species of the genus *Argiope*, such as *A. aemula, A. versicolor* and *A. keyserlingi*, web decorations that attract more prey also attract more predators such as wasps, mantises, or other spider predators (Cheng and Tso 2007; Seah and Li 2001; Bruce, Heberstein, and Helgar 2005). In *N. pilipes*, individuals with brighter coloration are more attractive both to prey insects and to predatory wasps (Fan et al. 2009). We stress here the necessity of considering sensory worlds of both prey and predators, as well as accounting for the fact that lures are multimodal and multicomponent. The review provided by White and Kemp (2015) on the sensory basis of color lures is, to our knowledge, the only attempt to date to incorporate a general sensory framework into the study of lures. This highlights a need for more mechanisms-oriented studies focused on explaining the sensory basis underlying the ability of spiders to produce efficient lures.

Given a plausible trade-off between prey attraction and predator avoidance, we should expect lures and other traits important to prey attraction to remain suboptimal despite a potential to reach greater attractiveness. Species using prey-attraction strategies might then seek to use lures that cannot be detected by their own predators. We suggest that this may be the case for highly specialized lures, such as the chemical components used by bolas spiders (Eberhard 1977; Stowe et al. 1987; Haynes et al. 2002). These lures, because they are specific to one (or a few) species of prey, are less likely to be detected or at least to attract predators. More generally, we urge future work to jointly address the sensory perceptions of both prey and predators when considering the costs and benefits of lures. In so doing, studies will help determining how attraction of both prey and predators operates and whether mechanisms that are central in signaling, such as learning (Guilford and Dawkins 1991, 1993) or key perceptual and cognitive abilities (Osorio and Vorobyev 2008), underlie sensory perception of spider lures. Meanwhile, prey-attracting spiders provide a useful system for comparative studies testing the possible role of coevolution in the diversification of sensory organs and receptors in signalers and receivers (Endler 1992).

## Potential evolutionary outcomes

One possible evolutionary outcome to which predator–prey interactions with lure-using predators may lead is to highly specialized lures. This scenario is generally expected when prey impose selection for morphological or physiological specialization in predators, constraining the predator to an extremely limited range of prey (Pekár and Toft 2015; West-Eberhard 2003; Begon and Townsend 2020). Any new adaptation that increases the predator’s efficiency to catch and consume prey then leads to new adaptations to avoid or escape the predator (Janzen 1980; Thompson 1989). The increased specialization of predators can also favor the coexistence of competing predator species as specialization allows resource partitioning and reduces the magnitude of competition among species (Miller et al. 2005). A particularly illustrative example of specialization in prey-attracting predators are bolas spiders, which attract only a limited number of prey species (Haynes et al. 2002). In theory, predators should often be selected to either become more specialist or more generalist depending on the extent to which capturing one prey species reduces the chance of capturing a different prey species (e.g., Abrams 2006).

In addition to the trade-off between prey attraction and predator avoidance, traits used for prey-attraction strategies can play a role in other functions and, as such, might be linked to life-history traits. Although this issue has rarely been investigated, there is some evidence that body coloration can affect both prey attraction and sexual selection. For example, white stripes of males of the spider *Dolomedes raptor* plays a role in both prey attraction and mate choice (Lin et al. 2015). The presence and size of white stripes in males, which depend on body size and presumably reflect resource acquisition during juvenile growth, have a positive effect on both prey attraction and female mating acceptance (Lin et al. 2015). The presence and intensity of the visual signal, such as the white stripes in *D. raptor*, might reliably indicate quality of potential sexual partners. Alternatively, this signal may have been initially selected for its role in signaling male quality to females and, once evolved, fortuitously contributed to prey attraction. To date, the specific mechanism underlying the origin of dual functions between prey attraction and sexual selection is unknown. Nevertheless, resolving the links between lures and life-history traits, and the subsequent overlaps between prey attraction and other functions, will certainly provide important insights into the evolution of prey-attraction strategies.

The trade-off between prey attraction and predator avoidance places a limit on the detectability of lures by prey. This is established for signals that are used in different contexts, such as in mate attraction and courtship. For example, in the mantis *Pseudomantis albofimbriata,* the only conspicuous part of this otherwise cryptic species is the achromatic (i.e., shades of gray) brightness of the abdomen of females, which is used as a signal of quality to conspecific males (Barry et al. 2015). This signal is associated with little risk of perception by eavesdropping predators, and may even improve camouflage through disruptive coloration (Barry et al. 2015). Although spiders can be expected to display the same strategy, spider lures differ from intraspecific reproductive signals in that they do not target conspecific receivers but a wide array of potential prey with different sensory abilities (Ximenes and Gawryszewski 2019). For instance, yellow lures are widespread presumably because this color is a highly efficient stimulus for many insect species (Craig 1996). Thus, lures function by covering a large range of sensory preferences. This implies that restricting this range to a more limited one, like mantises do, would almost certainly impede its efficiency in capturing prey. However, this is possible if one type of prey is predominant around the spider’s location as specialization would allow, and even favor, more restricted sensory ranges, and offer the opportunity to reduce detectability by predators. In the spinybacked orb-web spider *Gasteracantha cancriformis*, yellow morphs, which are attractive to every prey and predator taxa, coexist with red morphs, which mainly attract butterfly prey and bird predators but are inconspicuous for fly prey and wasp predators (Ximenes and Gawryszewski 2019). Polymorphism here would be both a product of the sensory landscape of prey and an adaptation to the sensory landscape of predators.

Although lures can in principle evolve to become highly attractive to prey while remaining cryptic to predators, spiders often forage on diverse prey and face diverse predators whose sensory capabilities are likely to overlap to some extent. This is suggested by studies which simultaneously compare the visual abilities of prey and predators, and show that contrasts and reflections attractive to prey also are detectable by a variety of predators, including invertebrate and vertebrate species (e.g., Heiling et al. 2005; Bruce and Heberstein 2005). These findings provide a mechanistic explanation to the widespread attractiveness of lures for both prey and predators. The decreased detectability of the *G. cancriformis* red morph for predatory wasps and dipteran prey can also be explained by the limited capacity of these insects to see in red wavelengths (while bird predators likely perceive them), whereas the brightness of the yellow morph matches best their visual abilities (Ximenes and Gawryszewski 2019). The overlap between predator and prey sensory abilities might be widespread as suggested in other contexts, such as in mimicry: species that are cryptic to their prey often are cryptic to predators too (Oxford and Gillespie 1998; Théry and Casas 2002; Théry et al. 2005; Defrize et al. 2010). For example, ambush crab spiders are known to be simultaneously cryptic in the color-vision systems of both bird predators and hymenopteran prey (Théry and Casas 2002). In the context of prey attraction, the presumably common overlap in predator–prey sensory abilities suggests that a trade-off generated by simultaneous prey attraction and predation risk must be widespread. Technics to model visual systems and contrast, such as those developed to study the sensory abilities of predators and prey in mimicking spiders (Théry and Casas 2002; Théry et al. 2005), warrants future progress in understanding how lures are perceived by both predators and prey.

An evolutionary solution to overcome the risk of being perceived by predators is to also exploit the sensory preferences of predators. For example, bright colors in *G. cancriformis* are detectable but unattractive for its predators, especially since birds associate bright colors such as red with unpalatability (Ximenes and Gawryszewski 2019; Brandley and Johnsen 2016). Similarly, the Australasian coin spider *Herrenia multipuncta* exhibits black and orange patterns that attract prey approaching from the dorsal side of the spider and deter predators approaching from the ventral side (Liao et al. 2019). Spiders of this species also adjust the visibility of a given side depending on the context, in response to the presence of prey and/or predators (Liao et al. 2019).

In general, the perception and preferences of prey might be important determinants of the evolution and maintenance of polymorphism in spider lures. This is evidenced by some species displaying lures that have multiple morphs (e.g., Kemp et al. 2013; Rao et al. 2015; White and Kemp 2016). This polymorphism in lures may be shaped by evolutionary constraints acting on sensory traits and driven by the preference of local prey communities (Craig and Ebert 1994; Kemp et al. 2013). Hence, polymorphism may be the result of the wide diversity in the visual perceptions of the various potential receivers, as well as environmental visual aspects impacting the visibility and attractiveness of lures, such as the color of the flowers in the local habitat and local light conditions (Craig et al. 1996; Kemp et al. 2013; White and Kemp 2015, 2016). Sensory traits in prey are potent agents of selection on lures, and might contribute to the maintenance of variation in prey-attraction traits within- and between species. Likewise, predators can contribute to the maintenance of polymorphisms in prey (Allen 1988; Merilaita 2006; Franks and Oxford 2017). And vice versa, variation in prey abundance can contribute to the maintenance of phenotypic variation in predators (Abrams 2006). Therefore, while this hypothesis has yet to be tested, variable predation pressure and prey abundance might contribute to the maintenance of variation in prey-attraction traits.

## Phenotypic variation in prey attraction

### Phenotypic plasticity

There is good evidence that phenotypic plasticity plays an important role in explaining variation, particularly in web decorations (e.g., Craig et al. 2001; Seah and Li 2002; Gawryszewski and Motta 2012; Llandres et al. 2011). Web decorations often vary over the lifetime of individual spiders that plastically adjust their strategy or simply respond to changes in their environment (e.g., Blackledge 1998b; Herberstein et al. 2000; Craig et al. 2001; Tso 2004). Spiders often adjust web decorations depending on changes in environmental conditions such as prey availability (Blackledge 1998b), predator presence (Bruce et al. 2001), and light and temperature conditions (Elgar et al. 1996; Herberstein and Fleisch 2003). For example, Herberstein and Fleisch (2003) showed that the orb-web spider *Argiope keyserlingi* tends to reduce the number of decorations when building a web at higher temperatures and increase the size of the decorations under lower levels of light. This increase in the visual signal might allow spiders to maintain foraging success when prey are less abundant at lower temperatures, or when prey are less likely to perceive visual signals in dimmer light.

Phenotypic plasticity, in addition to color polymorphism due to genetic differences, likely plays a role in the variation of body color used by prey-attraction species. This is because body coloration in spiders commonly varies with diet and environmental conditions during development (Oxford and Gillespie 1998). Nevertheless, it is still unclear to what extent phenotypic plasticity contributes to variation in body coloration of prey-attracting spiders. One possible reason for the lack of knowledge on this topic is that body coloration, unlike web decoration which is a labile trait, is likely determined during development and remains fixed during the adult lifetime of a spider. However, body color can change rapidly even in adult spiders in response to variation in environmental conditions. For example, body coloration changes through time, while color contrast changes with food intake in crab spiders (Gawryszewski et al. 2012). There is, thus, a need for more work to test for plasticity of body color by, for example, rearing spiders from different populations of a polymorphic species under identical laboratory conditions in a common garden experiment.

### Individual variation

Although intraspecific variation in web decorations is often the result of phenotypic plasticity in response to changes in environmental conditions (Craig et al. 2001; Gawryszewski and Motta 2012; Llandres et al. 2011), such variation can also reflect variation across populations and individuals of the same population (e.g., Kerr 1993) as the result of genetic differences (e.g., Craig et al. 2001; Nakata 2009). For example, a laboratory-based study showed that genetic variation explains part of the variation in web decorations in *A. argentata*. Nevertheless, little is known about individual variation in web decorations and whether such variation is comparable to variation resulting from phenotypic plasticity in response to changes in environmental conditions. More work is therefore needed to test for differences in the number and size of web decorations at both the species and population levels. Future studies addressing geographic variation in web decoration or individual variation within a single population would certainly provide important insights into local adaption and potential genetic differences in prey-attraction traits.

Body coloration of prey-attracting spiders, such as orb-web spiders of the genus *Gasteracantha*, provides a particularly well-known example of geographic polymorphism of traits associated with prey attraction. Populations of the Australian species *G. fornicata* tend to have stable morph frequencies along the gradient of distribution of the species, with the white morph prevailing in the north, and the yellow morph in the south (Kemp et al. 2013). The American species *G. cancriformis* also displays striking geographic variation in the number of spines and color, with at least 8 distinct morphs (Gawryszewski and Motta 2012; Salgado-Roa et al. 2018, 2022; Chamberland et al. 2020). Other taxa showing color polymorphism provide promising systems to study individual differences in prey attraction. This is the case, for example, in the giant wood spider *Nephila maculata* (Tso et al. 2002), the silver garden orb-web spider *Argiope argentata* (Craig and Bernard 1990; Craig and Ebert 1994; Craig et al. 2001), or the neotropical orb-web spider *Verrucosa arenata* (Rao et al. 2015). The mechanisms that generate and maintain color polymorphism are still debated, but likely involve adaptation to local environmental conditions or frequency dependence (Oxford and Gillespie 1998). There is in fact contrasting evidence showing that color morphs sometimes achieve greater capture rates in distinct habitats (e.g., Tso et al. 2002; Nakata and Shigemiya 2015; Rao et al. 2015), while in other species variation in body color has no effect on foraging success (e.g., Gawryszewski and Motta 2012; Llandres et al. 2011).

There is also some evidence showing that chemical signals used in prey attraction can vary among individual spiders. For example, the composition and the amount of web-bound putrescines used by orb-web spiders to attract prey to the web (Henneken et al. 2017a) vary among individual spiders and in response to changes in environmental conditions such as diet (Townley et al. 2006; Henneken et al. 2017b).

### The origin and maintenance of variation in prey attraction

The trade-off between attracting prey and avoiding predators may play an important role in maintaining variation in prey-attraction tactics. This is because, by imposing divergent selective pressures, the trade-off can also contribute to maintaining polymorphism within species and populations (Gray and McKinnon 2007; McKinnon et al. 2010). Meanwhile, the ability to produce alternative phenotype through polyphenism or behavioral responses can provide mechanisms to mediate opposing demands associated with attracting prey and avoiding predators (e.g., Van Buskirk et al. 1997; DeWitt et al. 2000; Eklöv and Svanbäck 2006). Thus, opposing pressures of attracting prey and predators may explain the maintenance of variation in lures. Phenotypic plasticity can allow spiders to cope with changes in the environment by readjusting their strategy to best match new environmental conditions. This is the case in species that adjust their web decoration according to the risk of predation by, for example, spinning fewer decorations on webs located in dense vegetation where predators have greater access (Bruce et al. 2001). Given costs of attracting prey, such strategies must be condition dependent, whereby only individuals that have access to enough resources and/or are in better condition can afford the costs. In general, heritable variation is crucial as it “fuels evolution,” and understanding the source of variation in prey-attraction strategies warrants insights into past and current evolution of these strategies.

Our knowledge about within- and between-individual variation in prey-attraction strategies depends heavily on the type of trait. As detailed above, phenotypic plasticity in web decorations has been well documented, whereas little is known about the developmental plasticity of body color in spiders using prey-attraction strategies. In contrast, there is good knowledge about color polymorphism and how body color varies among populations or among individuals within the same population, whereas little is known about geographical and among-individual variation in web decorations. Thus, to reduce this bias in the literature on phenotypic variation in prey-attraction strategies, we need more studies testing for (1) geographic variation in the frequency and use of web decorations, (2) repeatability in web decoration produced by individuals of the same population, and (3) plasticity of body color using common garden experiments.

## Conclusion

**Fig. 1:**
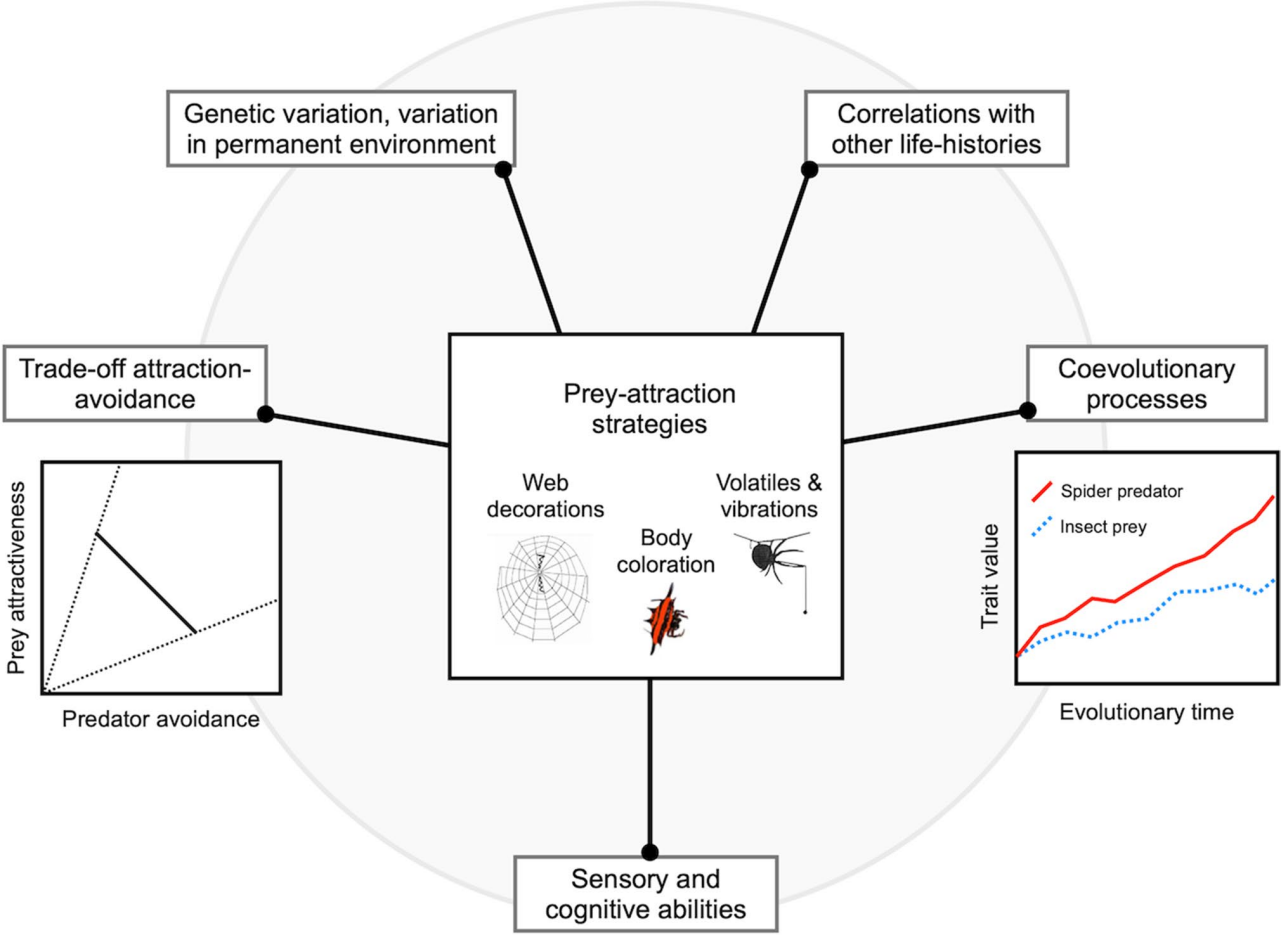
Summary of the main mechanisms constraining the evolution of prey-attraction strategies in spiders

In this review, we have shown that the evolution of prey-attraction strategies must be driven by the interplay of multiple environmental and intrinsic aspects of the organisms (Fig. 1). We have highlighted the key role that a trade-off between prey attraction and predator avoidance might play in driving predator–prey interactions in species that use prey-attraction strategies to forage. This trade-off is expected given that predators should seek to maximize prey capture while reducing predation risk. We have highlighted the importance of the manner that both predators and prey perceive their world in shaping the potential trade-off. We have also suggested that the opposing demands imposed by the trade-off can favor variation within populations and the maintenance of polymorphism, polyphenism, or behavioral plasticity. Yet, the trade-off has been the focus of relatively few studies, and we stress the need for more work addressing the occurrence and importance of the trade-off. Prey attraction is a special type of adaptation involving multicomponent signals, trade-offs, and correlational selection, and studying such complex traits would contribute to our understanding of the evolution of multivariate phenotypes in general.

**Fig. 2:**
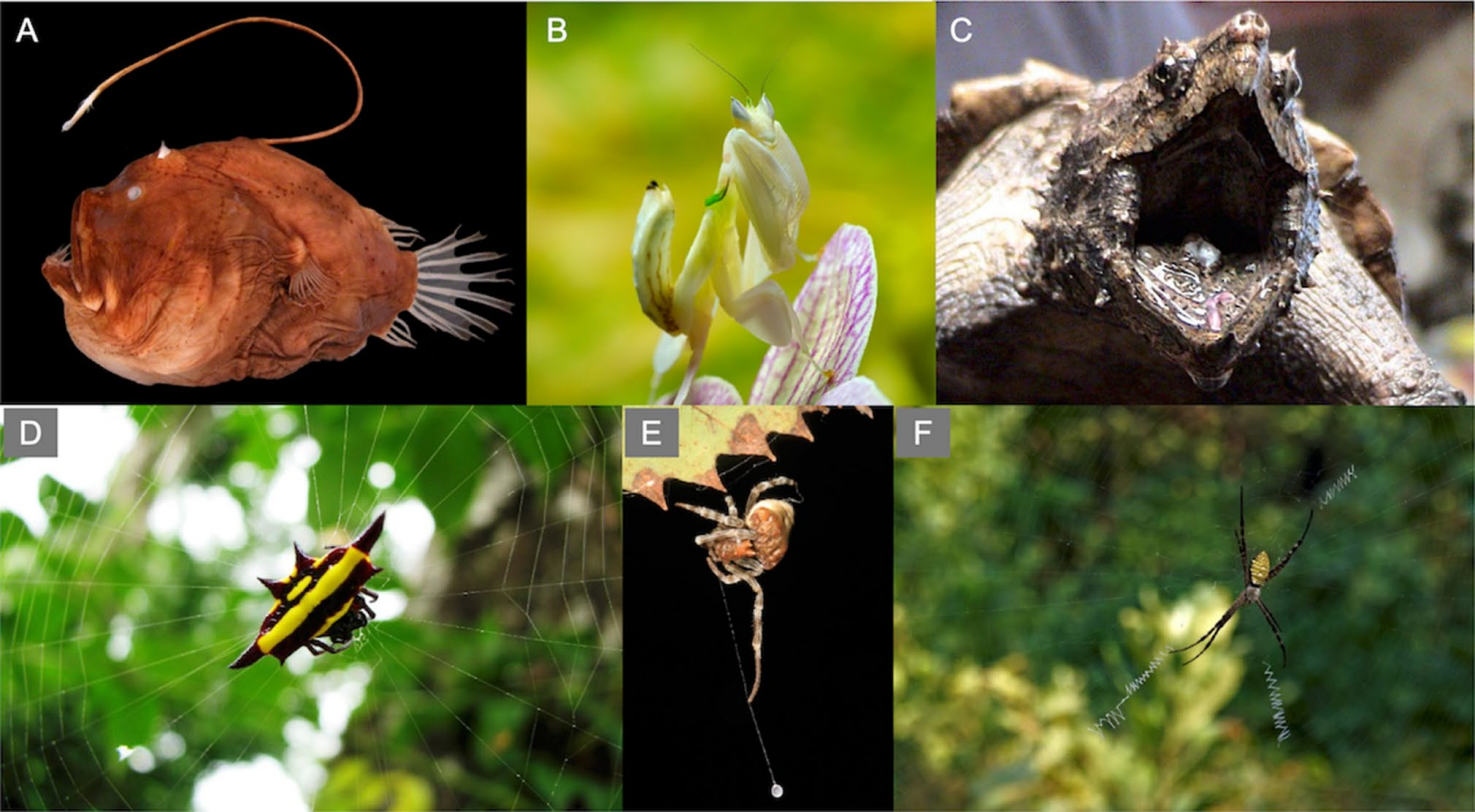
Prey attraction strategies in animals. **A** The deepsea anglerfish *Bufoceratias wedli* displays a large lure on its back and a smaller one located towards the front (image by Masaki Miya et al. 2010, CC BY 2.0). **B** The orchid mantis *Hymenopus coronatus* attracts and captures wild pollinators thanks to its body shape and color (image by Luc Viatour, CC BY 3.0). **C** The alligator snapping turtle *Macrochelys temminckii* uses its lingual appendage to imitate a small worm or an insect larva to attract its prey (image by LA Dawson, CC BY 2.5). **D** The northern jeweled spider *Gasteracantha fornicate* lures its prey using its conspicuous body coloration (image by Stephanie Levy, CC BY 2.0). **E** The bolas spider *Mastophora phrynosoma* lures male moths by producing a chemical mimicking female moth sex pheromone (image by Julie Metz Wetlands, CC BY 2.0). **F** The orb-web spider *Argiope aemula* decorates it web with stabilimenta to lure prey (image by Yagnesh Desai, CC BY 4.0)

There are multiple outstanding questions about prey-attraction strategies that are largely unsolved. First, we know little about the overlap of lures with other life-history traits, such as traits playing a role in reproduction. For example, in species where body coloration plays a role in both sexual selection and prey attraction, there may be opposing selection forces shaping body coloration. Alternatively, body coloration could fulfill the two functions, which seems to be the case with the white coloration in the nocturnal spider *Dolomedes raptor* (Lin et al. 2015). Second, although previous studies have documented the ecological cost in terms of increased predation, little is known about the physiological and/or energetic cost of producing lures or attracting prey in general. Overall, we should expect metabolic expenditures associated with the production of efficient signals for both prey and predators (e.g., Liao et al. 2019). There is, thus, a need for more studies addressing the cost of prey attraction. We hope to stimulate research on this topic and progress in our understanding of predator–prey interactions and, more generally, interactions among organisms Fig. 2.

Box 1 Evidence for prey attraction by predators Prey attraction in vertebrates is rare but is known in a diversity of species. For example, sidewinder rattlesnakes (Reiserer and Schuett 2008), vipers (Heatwole and Davison 1976), rat snakes (Mullin 1999), and saltmarsh snakes (Hansknecht 2008) use conspicuous body parts or coloration to attract prey. This is also the case in alligator snapping turtles (Spindel et al. 1987; Fig. 2), leaf frogs (Bertoluci 2002), toads (Hagman and Shine 2008) and anglerfish (Pietsch and Grobecker 1978; Fig. 2). In mammals, prey-attracting strategies have been suggested in the Arctic fox, where individuals modify the habitat near their dens in a way that attracts more prey (Gharajehdaghipour and Roth 2018). In these species, the visual signals may mimic food or potential mating partners, thus luring prey deceptively (Reiserer and Schuett 2008).

Apart from spiders where prey attraction is well known, prey attraction in invertebrates has been reported mainly in insects. At least two species of orchid mantises use their body coloration and shape to attract insect prey (O’Hanlon et al. 2014; Mizuno et al. 2014; Fig. 2), and larvae of a ground beetle lure frogs that mistake them for potential prey (Wizen and Gasith 2011). Females of a *Photuris* firefly prey on males of other firefly species by mimicking the flashing signals normally produced by conspecific females (Lloyd 1975, 1984). Some species of assassin bugs attract spider prey by generating vibrations on the web that attract the resident spider (Wignall and Taylor 2009, 2011).

## Data Availability

No new data were generated for this paper.
